# A *Plasmodium falciparum* genetic cross reveals the contributions of *pfcrt* and *plasmepsin II/III* to piperaquine drug resistance

**DOI:** 10.1128/mbio.00805-24

**Published:** 2024-06-24

**Authors:** John Kane, Xue Li, Sudhir Kumar, Katrina A. Button-Simons, Katelyn M. Vendrely Brenneman, Haley Dahlhoff, Mackenzie A. C. Sievert, Lisa A. Checkley, Douglas A. Shoue, Puspendra P. Singh, Biley A. Abatiyow, Meseret T. Haile, Shalini Nair, Ann Reyes, Rupam Tripura, Thomas J. Peto, Dysoley Lek, Angana Mukherjee, Stefan H. I. Kappe, Mehul Dhorda, Standwell C. Nkhoma, Ian H. Cheeseman, Ashley M. Vaughan, Timothy J. C. Anderson, Michael T. Ferdig

**Affiliations:** 1 Department of Biological Sciences, Eck Institute for Global Health, University of Notre Dame, Notre Dame, Indiana, USA; 2 Disease Intervention and Prevention Program, Texas Biomedical Research Institute, San Antonio, Texas, USA; 3 Center for Global Infectious Disease Research, Seattle Children’s Research Institute, Seattle, Washington, USA; 4 Mahidol-Oxford Tropical Medicine Research Unit, Faculty of Tropical Medicine, Mahidol University, Bangkok, Thailand; 5 Centre for Tropical Medicine and Global Health, Nuffield Department of Medicine Research Building, University of Oxford Old Road Campus, Oxford, United Kingdom; 6 National Center for Parasitology, Entomology and Malaria Control, Phnom Penh, Cambodia; 7 School of Public Health, National Institute of Public Health, Phnom Penh, Cambodia; 8 Boler-Parseghian Center for Rare and Neglected Diseases, University of Notre Dame, Notre Dame, Indiana, USA; 9 Department of Pediatrics, University of Washington, Seattle, Washington, USA; 10 BEI Resources, American Type Culture Collection (ATCC), Manassas, Virginia, USA; 11 Host Pathogen Interactions Program, Texas Biomedical Research Institute, San Antonio, Texas, USA; Washington University in St. Louis School of Medicine, St. Louis, Missouri, USA

**Keywords:** malaria, *Plasmodium falciparum*, genetic cross, piperaquine, drug resistance evolution

## Abstract

Piperaquine (PPQ) is widely used in combination with dihydroartemisinin as a first-line treatment against malaria. Multiple genetic drivers of PPQ resistance have been reported, including mutations in the *Plasmodium falciparum chloroquine resistance transporter* (*pfcrt*) and increased copies of *plasmepsin II/III* (*pm2/3*). We generated a cross between a Cambodia-derived multidrug-resistant KEL1/PLA1 lineage isolate (KH004) and a drug-susceptible Malawian parasite (Mal31). Mal31 harbors a wild-type (3D7-like) *pfcrt* allele and a single copy of *pm2/3*, while KH004 has a chloroquine-resistant (Dd2-like) *pfcrt* allele with an additional G367C substitution and multiple copies of *pm2/3*. We recovered 104 unique recombinant parasites and examined a targeted set of progeny representing all possible combinations of variants at *pfcrt* and *pm2/3*. We performed a detailed analysis of competitive fitness and a range of PPQ susceptibility phenotypes with these progenies, including PPQ survival assay, area under the dose response curve, and a limited point IC_50_. We find that inheritance of the KH004 *pfcrt* allele is required for reduced PPQ sensitivity, whereas copy number variation in *pm2/3* further decreases susceptibility but does not confer resistance in the absence of additional mutations in *pfcrt*. A deep investigation of genotype-phenotype relationships demonstrates that progeny clones from experimental crosses can be used to understand the relative contributions *of pfcrt*, *pm2/3*, and parasite genetic background to a range of PPQ-related traits. Additionally, we find that the resistance phenotype associated with parasites inheriting the G367C substitution in pfcrt is consistent with previously validated PPQ resistance mutations in this transporter.

**IMPORTANCE:**

Resistance to piperaquine, used in combination with dihydroartemisinin, has emerged in Cambodia and threatens to spread to other malaria-endemic regions. Understanding the causal mutations of drug resistance and their impact on parasite fitness is critical for surveillance and intervention and can also reveal new avenues to limiting the evolution and spread of drug resistance. An experimental genetic cross is a powerful tool for pinpointing the genetic determinants of key drug resistance and fitness phenotypes and has the distinct advantage of quantifying the effects of naturally evolved genetic variation. Our study was strengthened since the full range of copies of KH004 *pm2/3* was inherited among the progeny clones, allowing us to directly test the role of the *pm2/3* copy number on resistance-related phenotypes in the context of a unique *pfcrt* allele. Our multigene model suggests an important role for both loci in the evolution of this multidrug-resistant parasite lineage.

## INTRODUCTION

Malaria is a life-threatening parasitic disease that puts nearly half of the world’s population at risk (1). The World Health Organization (WHO) estimates that in 2020, there were 247 million malaria cases and 619,000 deaths reported across 84 countries, with 76% of those deaths occurring in children under the age of five (1). The continued evolution of drug-resistant *Plasmodium falciparum* asexual blood stage parasites has been one of the major obstacles to global malaria elimination and eradication efforts (1). Current frontline treatments for uncomplicated *P. falciparum* infection are artemisinin-based combination therapies (ACT), which are composed of a fast-acting artemisinin (ART) derivative paired with one or more longer-lasting partner drugs (2). ACTs began to replace historically efficient antimalarials, such as chloroquine (CQ) and sulfadoxine-pyrimethamine (SP), as first-line therapies in the early 2000s after resistance to these previous therapies swept throughout Africa (3–5). The WHO has recommended ACTs for uncomplicated malaria since 2006 due to their greater efficacy and decreased resistance compared with other single-drug therapeutics, with the most common ACTs used in Southeast Asia (SEA) being dihydroartemisinin/piperaquine (DHA + PPQ), artemether/lumefantrine (AL), and artesunate/mefloquine (AS + MQ). The ACT combination DHA + PPQ has been widely used throughout the Greater Mekong Subregion (GMS) of SEA, which has historically been a hotbed for the evolution of antimalarial drug resistance (6–8).

The spread of resistance to DHA + PPQ has been attributed to the rapidly expanding, multidrug-resistant KEL1/PLA1 lineage of parasites (9–11). These parasites originally emerged out of western Cambodia in 2008 and have since spread to Vietnam, Laos, and northeastern Thailand (10). KEL1/PLA1 parasites are characterized by ART-resistant mutations in *kelch13* (KEL1), specifically the C580Y substitution, as well as a copy number (CN) amplification of the two aspartic protease genes *plasmepsin II/III* (PLA1) associated with decreased susceptibility to PPQ (9, 10, 12, 13). Reduced susceptibility to the ART component of an ACT results in a greater remaining parasite load that has to be cleared by the partner drug, increasing the selective pressure for ACT failure (9, 14–17).

After the original detection of PPQ resistance (PPQ-R) in Cambodia in 2015, independent genome-wide association studies identified an association between *plasmepsin II/III* (*pm2/3*) variants and PPQ-R (16, 18–20). The mechanism proposed to support this association is an amplification of *pm2/3* that reduces concentrations of reactive heme in the parasite digestive vacuole (DV) to counter the inhibitory action of PPQ (20). The increased parasite survival rate at higher PPQ concentrations among Cambodian field isolates was associated with the amplification of *pm2/3* (13). Further *in vitro* studies of *pm2/3* amplification found that in the PPQ-sensitive (PPQ-S) 3D7 background, inactivation of *pm2/3* results in a mild increase in PPQ susceptibility, whereas overexpression of *pm2/3* alone did not impact the degree of parasite susceptibility to PPQ, artesunate (AS), or CQ (21, 22). Nevertheless, while *pm2/3* was initially heralded as a defining feature of PPQ-R, subsequent *in vitro* and population-based studies indicate that additional loci are needed to fully account for resistance (13, 23).

Beyond the role of *pm2/3*, decreased copies of *pfmdr1* and single nucleotide polymorphisms (SNP) in several other genes, including *exonuclease* (PF3D7_1362500) and the *P. falciparum chloroquine resistance transporter* (*pfcrt*), have been associated with PPQ-R parasites (16, 18, 24–27). Novel haplotypes containing amino acid substitutions in PfCRT were initially identified due to their increasing prevalence in areas utilizing DHA + PPQ (25). Additionally, *in vitro* editing in isogenic parasite lines demonstrated that these substitutions alone can impact parasite uptake of PPQ and drug susceptibility, independent of the *pm2/3* copy number (28–31). Field studies in the GMS report a continued expansion of some of these *pfcrt* haplotypes in the last 5 years that often co-occur in parasites carrying multiple copies of *pm2/3*, suggesting that these two loci are closely associated with each other (32, 33). Currently identified PPQ-R PfCRT substitutions (H97Y, F145I, M343L, G350R, and G353V) have been observed only in Dd2 or 7G8 CQ-R PfCRT backgrounds, suggesting possible constraints to their emergence (27, 34, 35).

Compared with other antimalarial drugs, assessing *in vitro* PPQ-R has been challenging due to the unusual dose response effect on PPQ-R parasites. These resistant parasites show incomplete killing at higher concentrations, resulting in an unusual bimodal dose response curve (13). To account for this non-traditional drug concentration-biological effect relationship, various modifications to standard IC_50_ values have been used, including area under the curve (AUC) (13). Alternatively, the PPQ survival assay (PSA) was developed to measure the parasite survival rate after exposure to a single pharmacologically relevant (200 nM) dose of PPQ (18). While these different readouts will correlate in their characterization of resistance, they also likely capture distinct aspects of the drug-parasite interaction. We predicted that genetic loci would contribute differentially to these different response readouts, perhaps pointing to distinct underlying biological significance in progeny inheriting unique allele combinations.

The genetic background of *P. falciparum* has been shown to play a central role in the emergence of drug resistance and its impact on parasite fitness (36–40). While several individual genes have been implicated in PPQ-R, the understanding of how these genes interact is lacking. The novel genetic recombination events resulting from a genetic cross could provide insights into how parasites develop resistance and how some lineages have strong potential to expand. To further dissect the contributions of different loci to PPQ-R, here, we examined progeny from a genetic cross between a multidrug-resistant Cambodian parasite of the KEL1/PLA1 lineage (KH004) and a drug-sensitive Malawian isolate (Mal31). At the time of isolation, DHA + PPQ (either with or without accompanied primaquine) had been used for treating uncomplicated *P. falciparum* malaria in Cambodia for over 15 years and was also used as the primary therapeutic for *P. vivax* infections. Additionally, the Mal31 parasite would have had no exposure to PPQ due to Malawi and neighboring countries solely utilizing AL as the primary therapeutic for uncomplicated infection. KH004 carries a C580Y Kelch13 substitution associated with ART-R, a Dd2-like PfCRT (74I, 75E, 76T, 220S, 271E, 326S, 356T, and 371I), associated with CQ-R, with an additional G367C substitution, and multiple copies of *pm2/3*. Mal31 is a wild type at *kelch13* and *pfcrt* and carries a single copy of *pm2/3* (Table S1). The G367C substitution in PfCRT carried by KH004 has previously been identified as a novel haplotype present in SEA. However, its association with *in* vitro PPQ resistance has not been determined (25, 34, 41).

Pairing a novel genetic cross with quantitative trait loci (QTL) mapping can be a powerful tool for quantifying the contributions of all loci to phenotypes of interest. We took a multiphenotype linkage approach to validate and characterize known resistance determinants as well as identify novel secondary loci associated with each unique resistance-related phenotype, underscoring differences in the biological features of these measured traits. We find that increasing levels of resistance to PPQ is prominently impacted by *pfcrt*, specifically a novel G367C substitution, with additional contributions from *pm2/3* amplification and other novel loci.

## RESULTS

### Experimental genetic crosses

We generated two biological replicate genetic crosses between Mal31 and KH004 using *Anopheles stephensi* mosquitoes and human liver-chimeric FRG huHep mice, as described by Vaughan et al. (42). There are 14,455 core-genome SNPs [defined by Miles et al. (43)] distinguishing these two parental parasites (44), and both parental parasites were cloned by limiting dilution. We generated the two recombinant pools for this cross using independent groups of 250 mosquitoes and intravenously injected salivary gland sporozoites from these two pools into separate FRG huHep mice. The oocyst prevalence rates from the two pools were 21% and 33%, respectively, and the average numbers of oocysts were 0.5 and 0.6. We were able to isolate approximately 225,000 salivary gland sporozoites from each pool, approximately 900 sporozoites per mosquito. Results from the parental feeds alone also showed a low oocyst prevalence (Mal31, 41% and KH004, 33%), with total sporozoite numbers per mosquito averaging 800 for Mal31 and 480 for KH004. The total number of potential recombinants from each pool (assuming 50% of oocysts are recombinant and 50% are parental) was 0.5 oocysts × 4 recombinants per oocyst × 250 mosquitoes × 0.5 = 250 for pool 1 and 0.6 oocysts × 4 recombinants × 250 mosquitoes × 0.5 = 300 for pool 2. The initial allele frequencies of Mal31 of the two recombinant pools directly from mice were 0.70 and 0.66, suggesting the existence of Mal31 selfed progeny.

We cloned and sequenced 567 progenies from the two recombinant pools (286 from pool 1 and 281 from pool 2) (Table S2). Initial analysis led to the removal of 153 non-clonal cultures (F_WS_ < 0.9 or non-clonal based on the allele frequency plot). Of the 414 clonal progeny cultures, 92 were selfed Mal31, 3 were selfed KH004, and 319 were recombinant. We identified a total of 104 unique recombinants [identical-by-descent (IBD) clusters], of which 65 were from pool 1 and 39 were from pool 2. There were 44 IBD clusters with more than two clones, and 60 were singletons. The results show a skewing toward selfing of Mal31, as predicted, and overall, 319/414 = 77% of the cloned progeny were recombinant.

### Determination of *plasmepsin II/III* copy numbers in parental parasites and progeny

**Fig 1:**
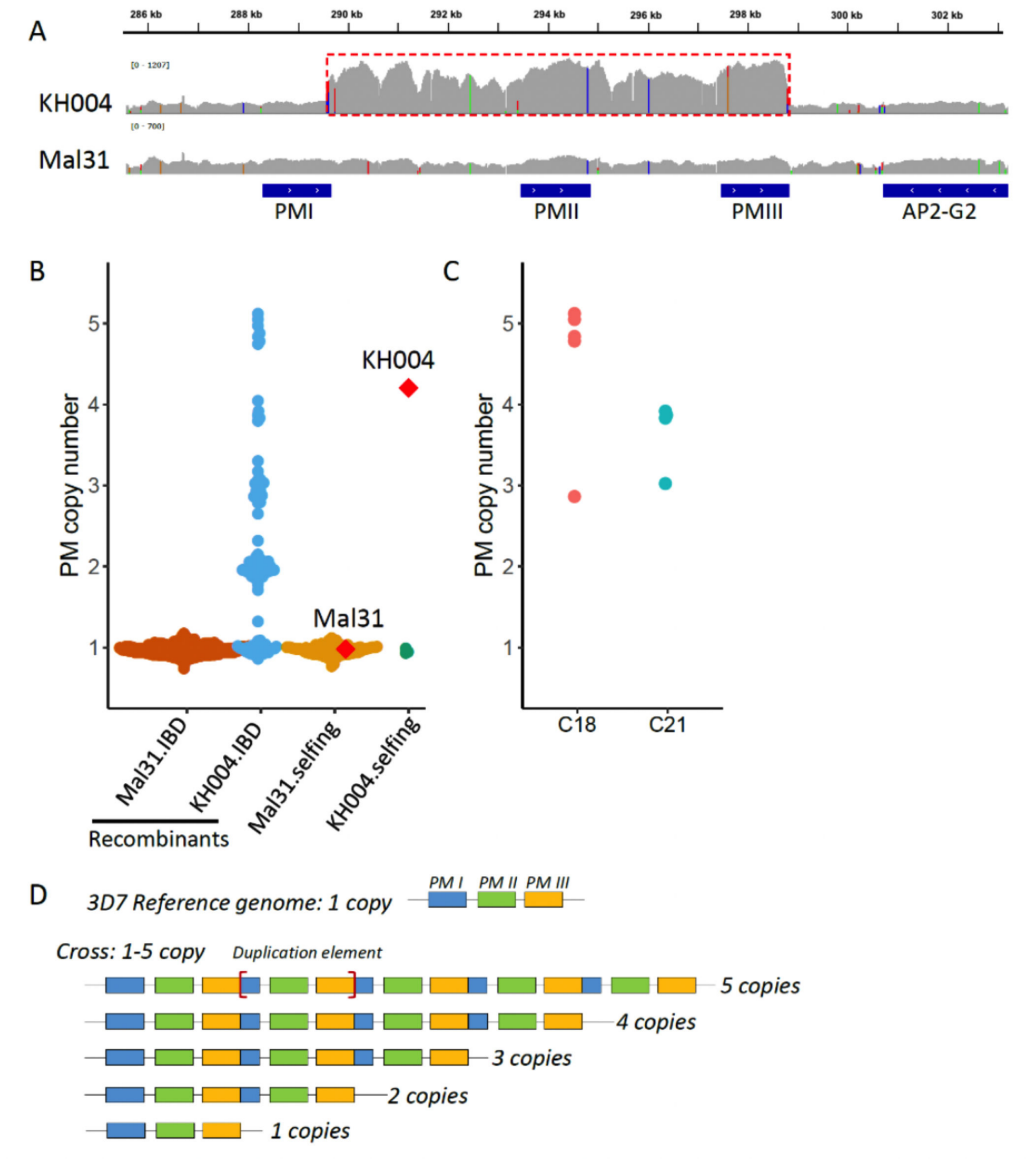
Plasmepsin II/III (pm 2/3) copy number variations in parents and progeny. (**A**) Integrative Genomics Viewer (IGV) plot showing Illumina short-read coverage at the pm 2/3 gene and flanking regions in parental parasites. The repeat unit of pm2/3 copy number variations was labeled with a red box. (**B**) pm2/3 copy numbers of parental parasites, recombinant progeny, and selfed progeny. Mal31.IBD indicates recombinant progeny with the pm2/3 allele inherited from Mal31; KH004.IBD represents progeny with pm2/3 from KH004. Parental parasites Mal31 and KH004 are labeled with red diamonds. (**C**) Rapid evolution leads to varying pm2/3 copy number in identical by descent (IBD) progeny. C18 and C21 are two IBD progeny groups, of which C18 contains five IBD clones, with four of them containing five copies and one of them containing three copies of pm2/3. C21 contains four IBD clones, with three of them containing four copies and one containing three copies of pm2/3. (**D**) Nanopore long reads from parental parasite KH004 that cover the pm2/3 copy number variation (CNV) and flanking regions. The boundaries of the repeat unit are labeled with and flanking regions. The boundaries of the repeat unit are labeled with (red square brackets).

We measured *pm2/3* CN for parental parasites with both nanopore long reads and Illumina short reads (Fig. 1A). Mal31 harbors one copy of *pm2/3*, while KH004 harbors multiple copies of *pm2/3*. We were able to extract four long reads from KH004 nanopore sequences that cover the *pm2/3* genes alongside >10-kb flanking regions (both upstream and downstream), and two of the reads had five copies, one had four, and one had three copies of *pm2/3*. The nanopore data demonstrate that the number of copies varies within the parental KH004 parasite population.

We further used Illumina short reads to analyze *pm2/3* CN in progeny (Fig. 1B and C), which revealed that *pm2/3* CN ranges from 1 to 5 in progeny; all selfed KH004 contained only one copy of *pm2/3*, in contrast to the parental KH004 that contained on average four copies of *pm2/3*; CNV of *pm2/3* was detected inside IBD clusters, i.e., C18 and C21 (Fig. 1C), which is consistent with CNV at *pm2/3* within the KH004 parental parasites.

### Assessment of novel allelic combinations of *pfcrt* and *pm2/3*

From our set of 104 unique recombinant progeny, parasites were grouped into 10 unique sets based on the inheritance of *pfcrt* and *pm2/3* alleles as well as *pm2/3* CN (Table S3). Importantly, progeny clones that inherited the KH004 *pm2/3* allele carry between 1 and 5 copies, with 28% (13/46) inheriting more than two copies. Among these progenies with more than two *pm2/3* copies, only 1 of 13 also inherited the KH004 allele at *pfcrt* (Table S3). In addition to suggesting that high *pm2/3* CN might reduce parasite viability, intensified by the co-inheritance of the resistant form of PfCRT, the range of CN associated with the KH004 *pm2/3* allele dampens the ability of QTL mapping to detect the allelic contribution of *pm2/3* CN to the measured phenotypes.

Genome wide, we observed two large skews toward Mal31 alleles, one on chromosome (chr) 7 and the second on chr 14 (Fig. S1). The skewed region on chr 7 is centered around *pfcrt*, which is known to carry high fitness cost with CQ resistance alleles (8). The apicoplast ribosomal protein S10 (ARPS10, PF3D7_1460900) is located at the peak of the chr 14 skewed region. Selection against the same *ARPS10* allele (V127M and D128H) has been previously detected in three other independent crosses between Asian and African parasites (27, 39, 45), indicating a strong fitness cost carried by this allele from an SEA parent (46).

We detected no skewed inheritance at *pm2/3*. We also saw no evidence for pairwise linkage disequilibrium or co-inheritance between *pfcrt*, *kelch13*, or *pm2/3* alleles. However, we did observe a significant (*P* = 0.006) association between inheritance of the KH004 ART-R *kelch13* allele and multiple copies of *pm2/3* (Table S4) as well as additional linkage between *pfcrt* and *arps10* (*P* = 0.011), which drive the skews on chromosomes 7 and 14, respectively.

### *In vitro* resistance phenotype of the KH004 parental parasite

**Fig 2:**
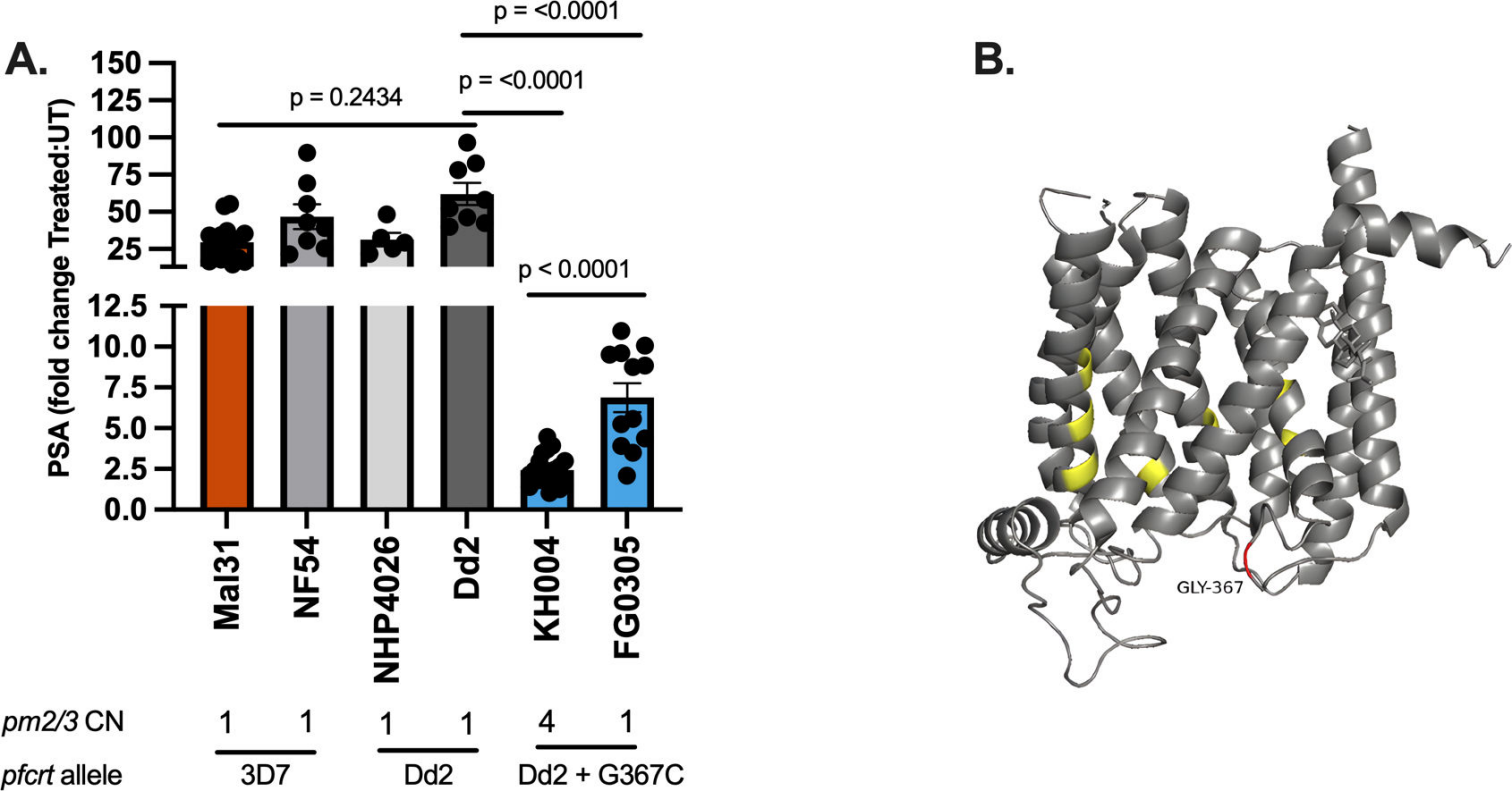
PfCRT G367C is associated with decreased PPQ susceptibility. (**A**) PSA between KH004, FG0305 (a selfed KH004 clone with single-copy pm2/3), two additional Southeast Asian parasites (Dd2 and NHP4026) that carry a Dd2-like PfCRT allele, and two African parasites (Mal31 and NF54) that harbor a wild-type 3D7-like PfCRT, identifies that the Dd2 PfCRT background alone is not sufficient for PPQ-R. The large fold change between treated and untreated samples (y-axis) indicates PPQ sensitivity observed for all samples lacking the G367C substitution. The smaller fold change in parasite lines with the G367C substitution indicates a reduced sensitivity to PPQ. This further supports the conclusion that PfCRT G367C contributes to PPQ resistance. Bars indicate mean ± SEM, and each dot represents a single biological replicate with three technical replicates. Statistical significance was determined via one-way analysis of variance and Mann-Whitney U tests. (**B**) AlphaFold model of Dd2 PfCRT with previously documented *in vitro* PPQ-R-associated mutations highlighted (yellow). Additional G367C amino acid substitution carried by KH004 (red) is in the DV-exposed portion of transmembrane domain 9.

KH004 carries a Dd2-like *pfcrt*, differing from Dd2 by only a G367C substitution (Fig. S2). Sequence identity between KH004 and Dd2 extends to the intergenic regions upstream and downstream of *pfcrt*. While the Dd2 *pfcrt* haplotype confers resistance to CQ and other antimalarials, it is not associated with PPQ resistance (25, 28–30). However, several studies have found that the addition of single PfCRT substitutions, specifically F145I, M343L, and G353V, into Dd2 is sufficient for significantly decreased sensitivity to PPQ (25, 28–30, 33, 34, 47, 48). The role of single PfCRT substitutions on PPQ sensitivity has also been observed in comparisons of natural isolates (49). Given the strong evidence for the role of PfCRT substitutions in PPQ-R, we suspect that the novel *pfcrt* genotype of KH004 is one of the major contributors to the observed resistance phenotype. Since this novel substitution has been recognized in areas of SEA with high PPQ administration but never directly assessed, we compared resistance phenotypes between KH004, a selfed version of this parasite (FG0305) which carries only one copy of *pm2/3*, Dd2, and NHP4026 [a recently isolated parasite from the Thailand-Myanmar border (46) that has a Dd2 *pfcrt* allele and one copy of *pm* 2/3] (Fig. 2A). PSA clearly shows that both KH004 and FG0305, independent of *pm2/*3 CN, are significantly less susceptible to PPQ, while those parasites without the G367C mutation (Dd2 and NHP4026) are highly sensitive to PPQ.

Additionally, comparisons between the isogenic parasites KH004 and FG0305 allow for us to isolate the contribution of *pm2/3* CNV in a KH004 pfcrt (PPQ-R) background (Fig. 2A). We find that while both single-copy and multicopy *pm2/3* versions of this parasite are significantly desensitized to PPQ, the original KH004 parasite which carries four copies of *pm2/3* is significantly less sensitive (*P* = 4.20*e*^−5^) than FG0305.

### PfCRT mutations are the major contributor to PPQ resistance

**Fig 3:**
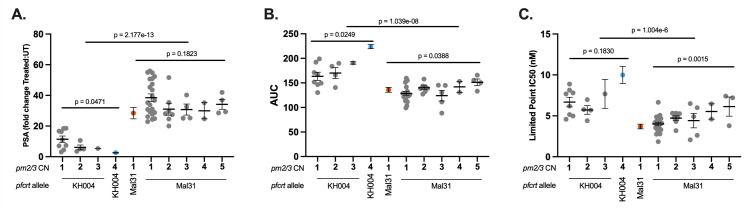
PfCRT mutations determine the majority of the variation in PPQ response. Progenies of the KH004 × Mal31 cross were grouped based on inheritance of parental allele at pfcrt (KH004 or Mal31) and pm2/3 copy number and were phenotyped using three different measurements of resistance: PSA (**A**), AUC (**B**), and LP-IC_50_ (**C**). We find that with all three phenotypes, inheritance of the KH004 pfcrt allele leads to significantly decreased susceptibility to PPQ. Additionally, we observed that increased copies of pm2/3 led to additional decreases in susceptibility; however, the impact of CNV with respect to pfcrt varied by phenotype. Parental parasites KH004 and Mal31 are designated in blue and orange, respectively. Bars indicate mean ± SEM, and each dot represents a unique recombinant parasite from our genetic cross. Linear models were utilized to assess statistical associations between PPQ phenotypes and both pfcrt inheritance and pm2/3 CN.

To comprehensively assess the impact of novel combinations of *pfcrt* and *pm2/3* on parasite response to PPQ, we measured three unique phenotypes: PSA, AUC, and a modified IC_50_ based on a limited-point dose response curve (LP-IC_50_) in both parents from the KH004 × Mal31 cross as well as 48 select progenies chosen to encompass all available combinations of *pfcrt* and *pm2/3* CN (Fig. 3).

IC_50_ is not traditionally used for phenotyping PPQ due to the atypical biphasic drug response curve generated by resistant parasites (13). We find that progenies that inherited the KH004 *pfcrt* allele and multiple copies of *pm2/3* generate a biphasic dose response curve, whereas progenies with only one copy of *pm2/3* or those that carry the Mal31 *pfcrt* allele produce a standard sigmoidal dose response (Fig. S3A). This observed range of dose response curve shapes within our progeny set affirms our use of multiple trait measurements, including a modification of standard IC_50_ values that rely on only the low concentration linear portion of the sigmoidal dose response (Fig. S3B) and the biphasic portion of the curve to calculate AUCs.

**Fig 4:**
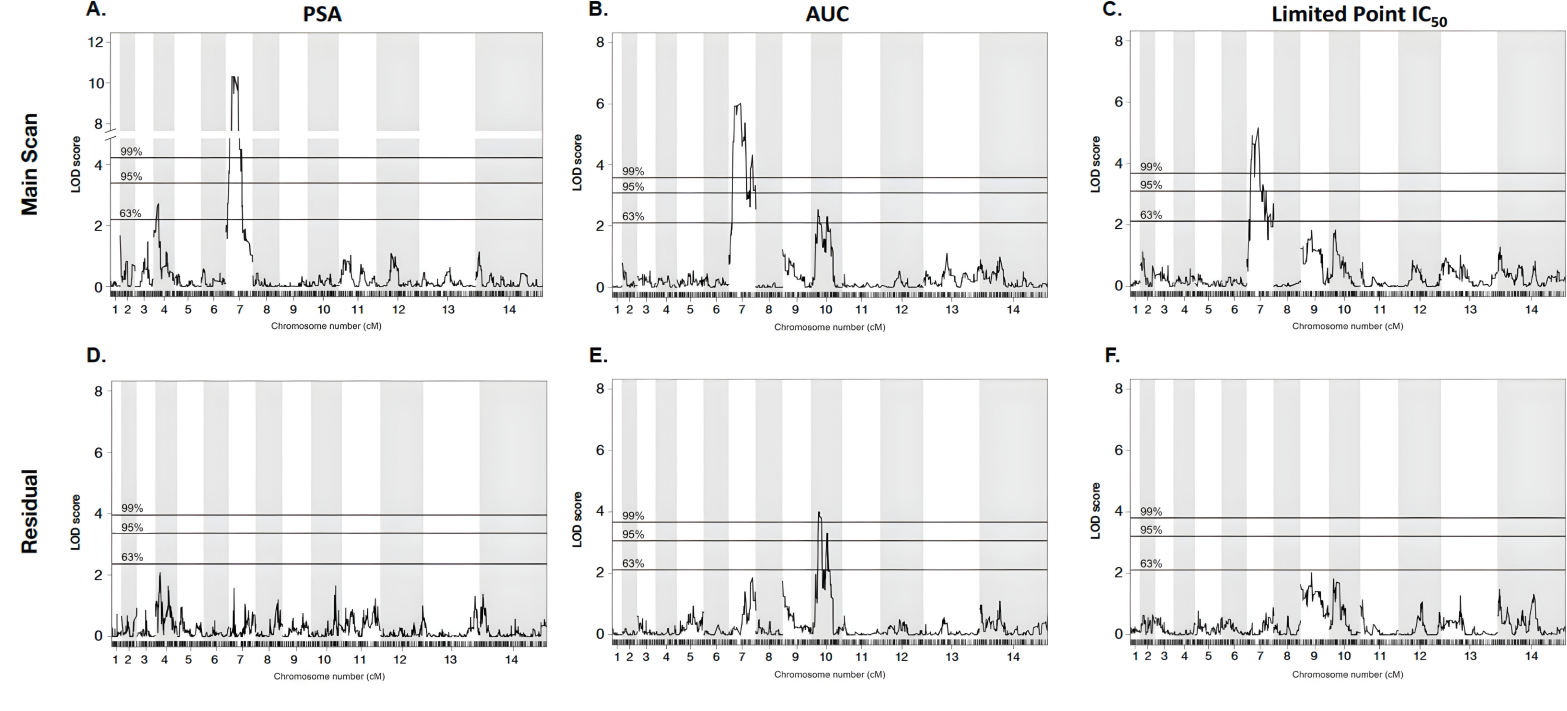
QTL mapping identifies major and secondary loci associated with PPQ-R. We performed QTL mapping based on parasite response to PPQ using our three metrics of resistance: PSA, AUC, and LP-IC_50_. For each of the three phenotypes (**A–C**), a region of chr 7 centered around pfcrt was most strongly associated with the variation associated with each phenotype. This further supports that pfcrt is the major factor associated with decreased susceptibility. To eliminate the potentially masking effect of the chr 7 QTL, residual scans of variation were performed (**D–F**). These scans identified a secondary QTL on chr 10 which was associated with AUC. Pairing QTL mapping with resistance phenotyping further supports the strong influence on pfcrt as well as identifies secondary contributors to PPQ-R.

We observe a significant association between *pfcrt* allele and PPQ susceptibilities, with *P* values of 2.2*e*^−13^ for PSA, 1.0*e*^−08^ for AUC, and 1.0*e*^−06^ for LP-IC_50_ (Fig. 3, Table S5). Additionally, QTL mapping of the PSA phenotype revealed a highly significant 334-kb region on chr 7 [logarithm of the odds (LOD) score of 10.3]] that centered on *pfcrt* (Fig. 4A). This region contains 82 genes, of which only 22 have non-synonymous mutations in the KH004 parent, which differ from Mal31 (Table S6). Of this subset of genes, a large portion has been uncharacterized, and *pfcrt* is the only gene that has previously been associated with decreased PPQ sensitivity. This strong peak is responsible for 62% of the observed inherited phenotypic variance in the PSA phenotype across the progeny set, whereas inheritance of this allele is responsible for 48% of the variance in the distribution of the AUC phenotype. QTL mapping of AUC and LP-IC_50_ also identify QTLs at this chr 7 locus (LOD scores of 6.9 and 5.2, respectively) (Fig. 4B and C).

### *pm2/3* CNV contributes epistatically to increased PPQ resistance

In addition to the major segregation of PPQ responsiveness based on *pfcrt* inheritance, we observe a significant association between *pm2/3* CN and PPQ susceptibility depending on phenotype and *pfcrt* inheritance (Fig. 3). We observe that *pm2/3* CN has a significant association with PSA in progenies that inherit the KH004 *pfcrt* allele (Fig. 3A). Conversely, LP-IC_50_ has a significant association with *pm2/3* CN only for progenies that inherit the Mal31 *pfcrt* allele (Fig. 3C). For AUC, we observe a significant association with *pm2/3* CN independent of *pfcrt* inheritance (Fig. 3B).

**Fig 5:**
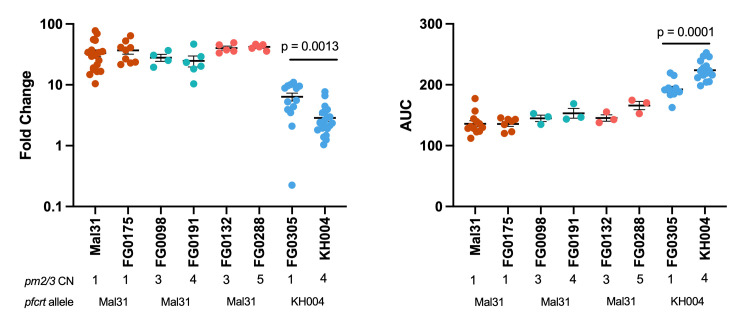
Effect of plasmepsin II/III copy number on PPQ-R is dependent on genetic background. Four groups of isogenic parasites which differ only in pm CN were assayed using (left) PSA and (right) AUC to identify the isolated role of pm2/3 CNV independent of genetic background. Of which, FG0175 is a selfed Mal31 progeny; FG0098 and FG0191 were isogenic through IBD (cluster C21) ; FG0132 and FG0288 are isogenic as determined by IBD (cluster C18); FG0305 is a selfed KH004 progeny clone. We found that CNV alone in a Mal31-pfcrt background does not significantly impact either phenotype. Bars indicate mean ± SEM, and each dot represents a single biological replicate with three technical replicates. Statistical significance was determined via Mann-Whitney U test.

To examine the role of *pm2/3* CN independent of other loci, we utilized two sets of parasites: isogenic (IBD) progeny and selfed parental parasites, which are genotypically identical except for differences in CN. These parasites, with the exception of the KH004 parent and its selfed version, all inherited the Mal31 *pfcrt* allele and phenotype as PPQ-S. Notably, we did not recover isogenic progeny with the KH004 *pfcrt* allele and varying CN. For both PSA and AUC, amplification from single-copy *pm2/3* to 4 copies in a PPQ-R (KH004 *pfcrt*) background significantly impacts both phenotypes; however, variation in the number of multiple copies did not significantly impact either phenotype in individuals expressing the Mal31 *pfcrt* allele (Fig. 5).

### Identification of additional loci contributing to PPQ response using QTL mapping

Due to the large and potentially masking effect of *pfcrt*, secondary scans of residual variation were examined for all traits after statistically removing the effect from the chr 7 locus (Fig. 4D through F). While no secondary effects were identified for either the PSA or LP-IC_50_ phenotypes, residual scans of the AUC phenotype identify a significant (LOD = 4.0) QTL on chr 10, which contains 61 genes (95% CI) (Table S7). Additionally, we used a two-dimensional, two-QTL genome scan to identify and characterize significant interactions between loci that contribute to the resistance phenotypes (Table 1). Using a two-locus model, we identified a significant additive interaction between loci on chr 7 and chr 10 (LOD additive: 10.7; 5% threshold) for the AUC phenotype. Conversely, no QTL interactions were identified using the PSA or LP-IC_50_ phenotypes. These results highlight how the different methods used to measure the PPQ response uncover different genetic interactions, underscoring the genetic complexity of the decreased susceptibility to PPQ seen in SEA parasite strains.

**TABLE 1:** Additive interactions influencing PPQ-R phenotypes^*a*^

Phenotype	Pairs of loci	Position locus 1	Position locus 2	LOD interaction (epistasis)	LOD additive
AUC	Chr 7: Chr 10	357253	485200	1.39	**10.7**

^*a*^ Two-dimensional genome scan identifies interacting loci on chromosomes 7 and 10 contribute to the AUC phenotype. We have identified that these two loci interact additively in their contributions to AUC. We identified no interacting loci associated with the PSA phenotype. Significant LOD scores are in bold and have surpassed the 5% threshold.

### PfCRT G367C is associated with decreased PPQ sensitivity

Comprehensive analysis of KH004 × Mal31 progeny points to *pfcrt* as the major determinant of PPQ resistance, along with modulatory effects from other loci. Inheritance of the *pfcrt* allele from KH004 was the major factor shared by PPQ-R parasites (Fig. 2 and 3). KH004 carries a Dd2-like *pfcrt*, differing from Dd2 by only a G367C substitution (Fig. S2). While the Dd2 *pfcrt* haplotype confers resistance to CQ and other antimalarials, it is not associated with PPQ resistance (25, 28–30). Thus, we suspect the G367C substitution is directly influencing the observed phenotype in KH004.

Reported drug resistance-associated mutations in PfCRT generally reside in one of the 10 transmembrane domains, specifically in regions that make up the central cavity of the transporter (34, 50). The G367C substitution is distinct from experimentally validated PPQ-R-associated mutations in that it resides in the DV-exposed portion of transmembrane 9 and is not part of the core structure of the transporter’s central cavity (28, 29, 34, 48, 51) (Fig. 2B).

**Fig 6:**
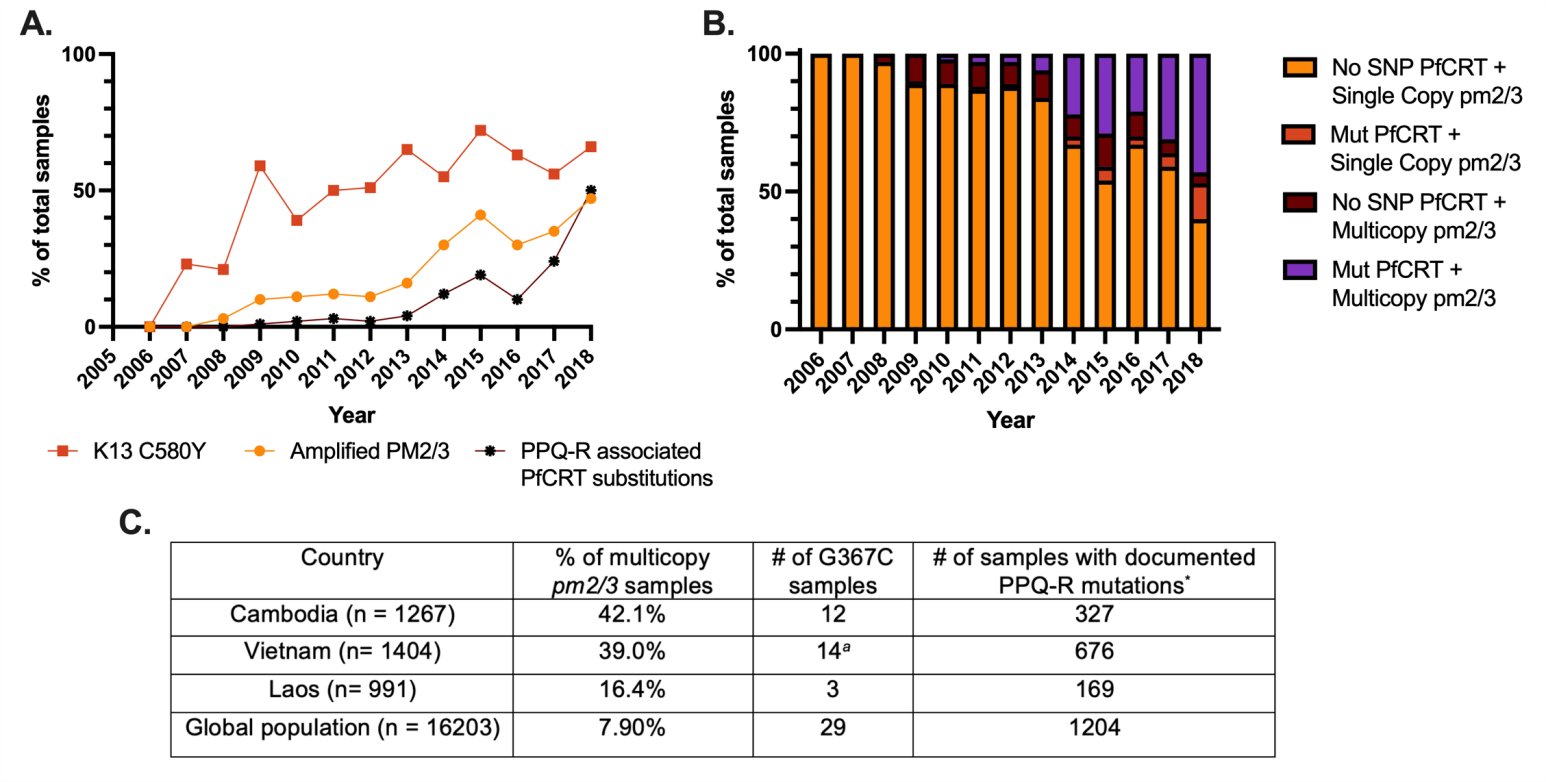
Increasing frequency of pm2/3 amplification and novel pfcrt mutations associated with PPQ-R in the GMS. Using available data from MalariaGEN Pf7 release (*n* = 3,359), samples from Cambodia, Vietnam, and Laos were analyzed for the association between pm2/3 amplification and novel PPQ-R-conferring pfcrt mutations. (**A**) The rise in novel pfcrt PPQ-R-associated genotypes immediately following amplification of pm2/3 further supports the important roles of both pm2/3 and pfcrt in the evolution of PPQ-R. (**B**) Most parasites carrying novel PPQ-R-associated pfcrt mutations also have multiple copies of pm2/3. (**C**) The novel G367C substitution has been observed throughout SEA, although at low levels, since 2012. Apart from two samples, this mutation is found in PPQ-R samples and is also accompanied by increased copies of pm2/3. ^a^Two samples from Vietnam carry a single pm2/3 copy and were categorized as sensitive due to single copies of pm2/3, without consideration of pfcrt polymorphisms. ^*^PPQ-R-associated PfCRT substitutions include T93S, H97Y, F145I, I218F, M343L, and G353V. The C350R substitution was omitted as it arose independently out of a 7G8 background and has not been identified on top of a Dd2 background.

This novel G367C mutation was noted by Ross et al. (25) to be present in SEA at low prevalence in a small geographic focus. Because the KEL1/PLA1 lineage has continued to spread throughout SEA, coinciding with the continuing emergence of PPQ-R, we analyzed the prevalence of this novel mutation over time using the MalariaGEN Pf7 data set. Of 16,203 global *pfcrt* haplotypes, 29 samples carried G367C (Fig. 6). This novel haplotype was first identified in 2012 and was restricted to Cambodia until 2016, but it has more recently emerged in neighboring countries Vietnam and Laos (Table S8). Furthermore, 93% of these 29 clinical samples also carry multiple copies of *pm2/3*. We also note the timeline of the emergence of these novel PPQ-R-associated pfcrt mutations in SEA: Kelch13-mediated ART-R mutations emerged first, while the rise of novel PPQ-R-associated *pfcrt* mutations quickly followed the increase of samples expressing multiple copies of *pm2/3* (Fig. 6). Interestingly, this trend appears to be unique to SEA, as Florimond et al. have reported that novel *pfcrt* mutations preceded *pm2/3* CN amplification in South America (49).

### Impacts of *pfcrt* and *pm2/3* inheritance on parasite blood stage fitness

Our data from multiple resistance phenotypes support that *pfcrt* plays the primary role in decreased PPQ sensitivity, with a boost to the degree of resistance resulting from increased copies of *pm2/3*. It has also been well documented that mutations in major resistance-associated genes can be associated with a loss in parasite fitness (31, 52, 53). Deleterious fitness effects of the KH004 *pfcrt* allele are evident from the reduced representation of this allele in the progeny recovered from the genetic cross (Fig. S1). For these novel resistance-associated genotypes to increase in global prevalence in the absence of selection from widespread drug treatment, parasites must preserve fitness either through low-cost resistance mutations or the acquisition of secondary compensatory mutations that preserve or improve fitness. We conducted competitive growth assays between progeny of the KH004 × Mal31 cross and both parental parasites to assess blood stage fitness differentials (Fig. S4). Progeny vs parent competitions revealed that most progeny had a relative fitness situated between the two parents, while four progeny parasites were outside of the parental fitness range (two were more fit than KH004 and two were less fit than Mal31), with no significant clustering in fitness based on inherited *pfcrt* and *pm2/3* genotypes (Fig. S4).

**Fig 7:**
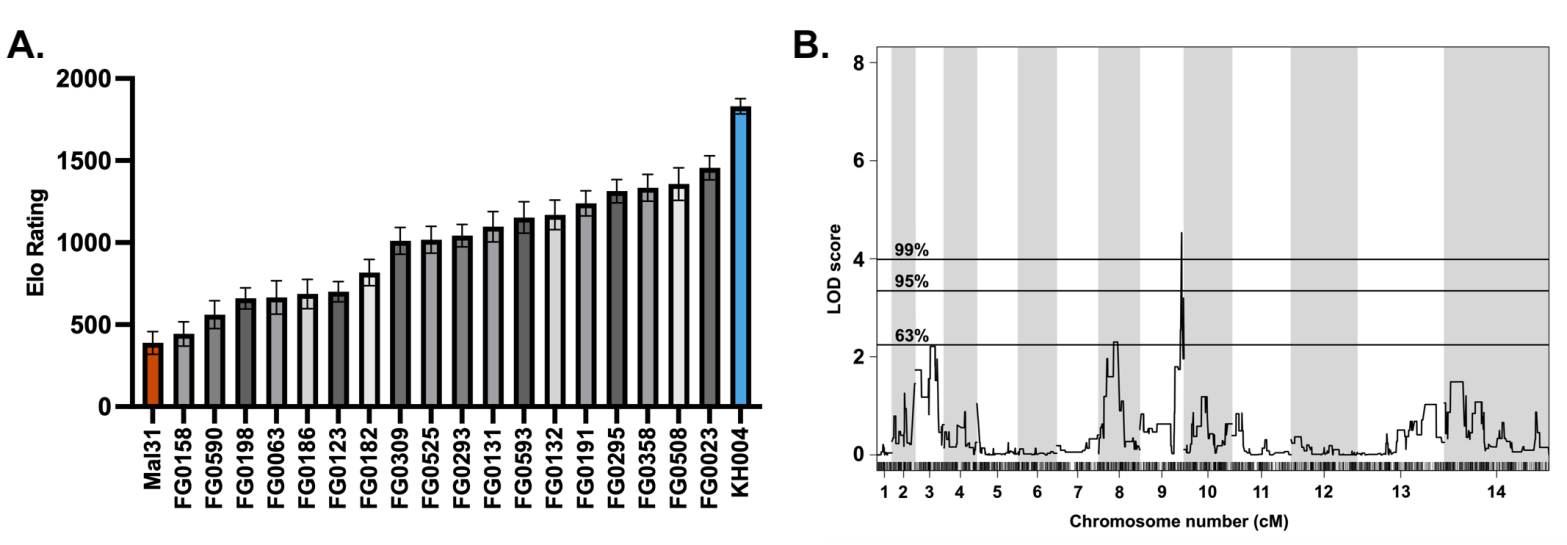
QTL mapping of fitness-associated loci using Elo rating phenotype. (**A**) Mean Elo rating ±SD of 18 progeny + 2 parents of the KH004 × Mal31 genetic cross based on 1,000 permutations of the order of competitions. Elo rating was calculated using the R package EloRating and was based on each parasite’s competitive growth assay outcome. Phenotype distribution is ordered from lowest Elo (least fit) on the left to highest Elo (most fit) on the right. (**B**) QTL scan based on Elo phenotype reveals a highly significant peak (1% threshold) on chromosome 9. This peak encompasses a 35,264-bp region which contains 15 genes. Genes identified from this QTL scan are proposed to directly contribute to parasite fitness.

Based on the similar fitness phenotypes generated from competitions against the parental parasites, a select subset of 18 progeny and both parents was set up in overlapping competitions against common competitors, resulting in 212 competitive outcomes (win, lose, or draw). The outcomes of these pairwise competitions were used to generate an Elo rating for each parasite using the *EloRating* package in R (54). Elo ratings provide a weighted ranking of parasite fitness based on competitive growth outcome as well as the fitness ranking of the competitor, with a higher Elo rating representing a greater competitive fitness level. Elo ratings for these 20 parasites (parents plus progeny) provide a much finer resolution of fitness differences between parasites of seemingly similar fitness. Unlike the competitions against both parents, Elo ratings provide a more continuous phenotype, but still do not cluster based on the *pfcrt* and *pm2/3* genotypes (Fig. 7A). However, QTL analysis using Elo phenotypes reveals a highly significant threshold (1%) on chromosome 9 (LOD score of 4.5), which encompasses a 35-kb region containing 15 genes (Fig. 7B). Parasites with the greatest competitive fitness were associated with the inheritance of this 35-kb region from the KH004 parent. Sequence comparison between KH004 and Mal31 for these 15 genes identified four non-synonymous coding SNPs in two genes: a

TFIIH transcription factor (PF3D7_0934100) and a second gene encoding a putative UBX domain-containing protein (PF3D7_0934700) (Table S9). Since the KH004 parent contains two SNPs in the TFIIH transcription factor and both KH004 and Mal31 have mutations in the second gene, it is unclear as to whether the combination of mutations acquired by the KH004 parent increases fitness or if the single mutation in the Mal31 parent leads to a fitness defect.

**Fig 8:**
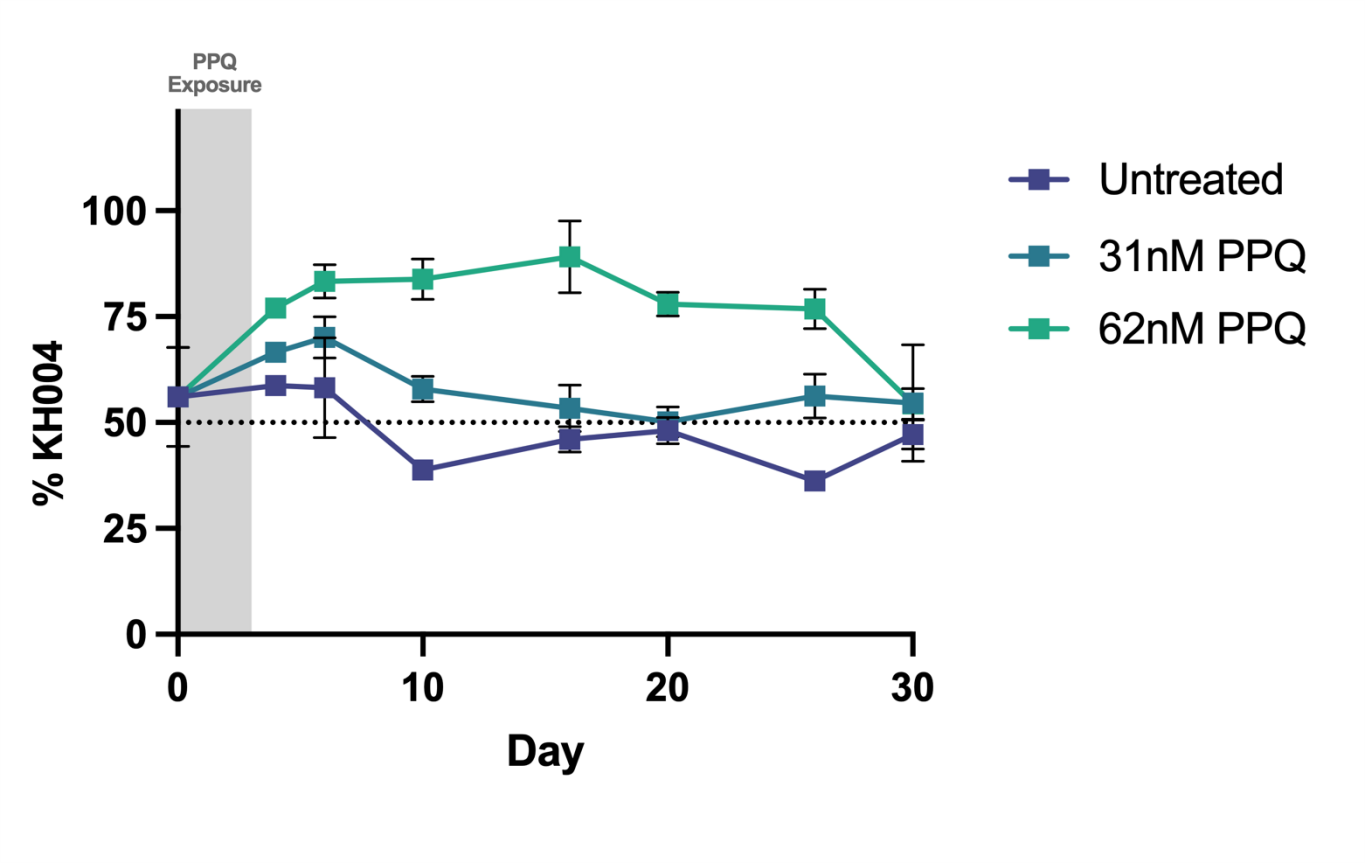
Impact of pm2 CN alone on fitness and PPQ susceptibility. Competitive growth assays were performed between isogenic parasites KH004 and FG0305 in the presence and absence of PPQ. KH004 has four copies of pm2/3 whereas FG0305 has a single copy. Treated samples were exposed to PPQ for 3 consecutive days following setup. We detect no impact on competitive fitness associated with changes in pm2/3 CN (untreated). Additionally, we observe the impact of increased CN on PPQ sensitivity, with moderate PPQ pressure selecting for the multicopy KH004 parasite. Percentage abundance of KH004 was determined by average number of pm2 copies in the co-culture, with four and one copies representing complete fixation of KH004 and FG0305, respectively, and 2.5 copies representing and equal ratio of each parasite. Each data point represents the mean ± SEM of three biological replicates, of which each setup had three technical replicates.

Additionally, to isolate the role of *pm2/3* in parasite fitness, we competed isogenic parasites KH004 (four copy *pm2/3*) and FG0305 (one copy *pm2/3*) in the presence and absence of PPQ pressure (Fig. 8). The outcome of this competition was measured by changes in total *pm2* CN of the mixed culture, with an increase in copies representing a greater abundance of KH004. In the absence of drug pressure, these two parasites remain in equal ratios over the course of 30 days. However, exposure to 31 nM and 62 nM PPQ selects for the multicopy KH004 parasite. After drug pressure was removed, all competitions eventually returned to equivalent amounts of each parasite. Thus, the *pm2/3* copy number plays a role in increased fitness in the presence of drug but no role in the absence of drug.

## DISCUSSION

In this study, we used a genetic cross between a multidrug-resistant Cambodian parasite and a drug-sensitive isolate from Malawi to characterize the genetic contributors to PPQ-R. Previous work on this subject has relied on naturally resistant parasites isolated from patients or *in vitro* experimental manipulations to test the ability of specific mutation to confer PPQ-R (13, 16, 20, 25). The use of a genetic cross allows us to track the inheritance and impact on phenotypes of alleles previously associated with resistance, such as SNPs in *pfcrt* or amplification of *pm2/3*, while also searching for additional trait-impacting loci across the genome. This classical genetic approach demonstrates that the inheritance of the KH004 PfCRT (Dd2 + G367C) is the main driver of PPQ-R, with the amplification of *pm2/3* providing additional phenotypic variation.

The KH004 parent of this cross has a novel G367C substitution in PfCRT, previously noted in SEA but not directly associated with PPQ-R (25). Through comparisons between KH004, Dd2, and other SEA parasites that have identical *pfcrt* genotypes to KH004 except for G367C, we observed a >30-fold change in PSA between KH004 and parasites expressing a standard Dd2-like PfCRT, indicating a direct connection between this substitution and PPQ-R. This association between PPQ-R and PfCRT substitutions has been well documented by genetically modifying single amino acids in this transporter (25, 29, 30, 34, 47). While the G367C substitution has not previously been connected to PPQ-R, this residue is within the binding site of the structurally similar chloroquine derivative perfluorophenylazide biotinylated chloroquine (AzBCQ) (55). The identification of this residue’s association with PPQ-R contributes to a broader understanding of the PfCRT structure that influences a wide range of drug susceptibilities, i.e., the range of *pfcrt* haplotypes shaped by evolution to drug pressure. The major *pfcrt* haplotypes associated with PPQ-R have all evolved on a 7G8 or Dd2 background, and transfection of these mutations into different *pfcrt* backgrounds has found that the Dd2 *pfcrt* genotype supports the highest level of PPQ-R compared with other backgrounds (29, 34).

Previously studied PPQ-R-associated PfCRT substitutions including T93S, H97Y, C101F, F145I, M343L, and G353V were suspected due to their increasing prevalence in sites using DHA + PPQ (25, 34). The novel G367C substitution expressed by KH004 arose in western Cambodia in 2012 and was found at a low abundance in the population. Since its initial emergence, the Dd2 + G367C haplotype has continued to persist at low abundance but has spread to neighboring countries, Laos and Vietnam, with the latter accounting for the majority of identifications in recent years. Whereas most of SEA has transitioned from DHA + PPQ to alternative combination therapies as frontline treatments, Vietnam continues to use PPQ, which might explain the recent increase in the abundance of this novel haplotype within the country (1, 56, 57). On the other hand, the introduction of this haplotype into Laos can potentially be accounted for by a recent introduction of PPQ-R parasites into the country from Cambodia (58).

Due to unusual PPQ-parasite interactions that are not effectively captured by standard dose response (IC_50_) curves, we utilized three unique phenotypes to illuminate different aspects of PPQ-R: AUC, LP-IC_50_, and PSA. By using these different assays, we deconstruct the resistance phenotype into three related but distinct biological readouts: survival under high single-dose PPQ (PSA), low-dose growth inhibition (LP-IC_50_), and shape of the dose response curve under high PPQ concentrations (AUC), to refine our search for the genetic factors controlling these phenotypes (13, 18, 21, 22). QTL Mapping for each trait identified mutant *pfcrt* as the main underlying contributor to resistance. Additionally, statistically removing the effect of this locus allowed for the identification of secondary contributors to the corresponding phenotype. While the PSA and LP-IC_50_ residual scans did not identify secondary loci, an additional locus on chr 10 was strongly associated with the AUC phenotype (Fig. 4). One of the genes with the strongest association within this region is *autophagy-related gene 18* (*atg18*). KH004 contains a single non-synonymous SNP in *atg18*, leading to a T38I substitution, whereas Mal31 carries the wild-type 3D7-like variant of this gene, highlighting a candidate mutation for further investigation. This protein localizes to the parasite DV and has been associated with decreased susceptibility to several antimalarials such as DHA, artemether, and PPQ, while also being connected to increased survival under nutrient deprivation (59–61). Due to its previous association with resistance to PPQ and other antimalarials such as lumefantrine and mefloquine, as well as association only with the AUC phenotype, *atg18* may contribute to the non-traditional dose response curve encountered when studying *in vitro* PPQ-R (27). While we have identified a combination of novel and previously reported loci through QTL mapping, the impact of additional genes associated with resistance to other antimalarials, such as pfmdr1 and pfaat1, on decreased PPQ sensitivity, cannot be determined due to genetic similarity between KH004 and Mal31 at these loci. Because inheritance of the KH004 *pm2/3* allele does not correspond to a specific number of copies and the majority of the progeny inherited a lower CN than the KH004 parent, we also do not detect the impact of *pm2/3* CN on PPQ susceptibility as a QTL (Fig. 4), despite our observation that increased CN is associated with decreased PPQ sensitivity in parasites which also inherit the KH004 *pfcrt* allele (Fig. 2 and 3). The association between increased CN and decreased PPQ susceptibility was also directly measured using isogenic parasites, which differ only in *pm2/3* CN (Fig. 5). The multicopy KH004 parasite showed decreased PPQ susceptibility compared with single-copy FG0305 for both AUC and PSA phenotypes (Fig. 5) and was also selected for under drug pressure when in co-culture with FG0305 (Fig. 8).

Previous studies hypothesized that instead of directly modulating PPQ-R, expressing multiple copies of *pm2/3* amplification may play a compensatory role in parasite fitness (22). This hypothesis, paired with extensive previous findings on the fitness costs of *pfcrt* resistance mutations, led us to assess the competitive fitness levels of our progeny with various combinations of *pfcrt* and *pm2/3* (30, 51, 52). By comparing progeny fitness to KH004 and Mal31 parents, we found no significant clustering of *pfcrt/pm2/3* genotype combinations that suggest a simple genetic determination of parent-derived fitness. Consequently, we leveraged all-on-all competitions to generate our novel implementation of Elo rankings, which can efficiently compare head-to-head fitness levels across a large progeny set and generate a refined phenotype for QTL mapping. Using this approach, we identified a highly significant association between parasite fitness and a narrow 35-kb region on chr 9. This region includes genes involved in development, transcriptional regulation, and metabolism, as well as two prioritized candidate genes with coding variations between KH004 and Mal31, a TFIIH transcription factor (PF3D7_0934100), and a second gene encoding a putative UBX domain-containing protein (PF3D7_0934700). Neither gene has been linked previously to parasite fitness. We performed a head-to-head competition between two isogenic parasites KH004 and FG0305, which only differ in *pm2/3* CN, to assess the fitness cost of having multiple copies of *pm2/3*. We find that changes in *pm2/3* CN alone do not impact parasite competitive fitness.

Regardless of the strong selection against the inheritance of the KH004 *pfcrt* allele in our progeny set, we find no evidence that carrying this allele comes at a fitness detriment with respect to our head-to-head competitive growth assays. The inheritance of this allele may present a greater fitness cost in multiparasite competitions, as would be the case in bulk pools of recombinant progeny prior to cloning or during other stages of the parasite lifecycle. Additionally, because of the selection against the mutant *pfcrt* allele, recovered progenies that inherited this have likely also undergone selection at other regions of the genome, to compensate for this fitness cost. This lack of correlation between inheritance selection and competitive fitness has also been observed in our previous genetic cross: 3D7 × NHP4026 (40). In this cross, NHP4026 has a Dd2 CQ-R *pfcrt* genotype, and inheritance of this allele is strongly selected against, yet it is fit (Fig. S5). This shift in fitness cost between allele inheritance and *in vitro* measures of parasite fitness highlights the importance of genetic background, which can support the inheritance of alleles that are selected against. Importantly, while KH004 showed the highest level of fitness in this cross, it has a moderate fitness phenotype compared with other well-studied *P. falciparum* parasites (Fig. S5).

Deeper knowledge of the genetic determinants of PPQ resistance can point to novel control and prevention strategies. Our classical genetic approach informed our understanding of the major drivers of resistance, including a novel *pfcrt* mutation, and also identified secondary genetic factors that contribute to parasite resistance and fitness. Incorporation of a range of PPQ-R phenotype measures into our analysis revealed unique genetic regions and their interactions associated with changes in each phenotype. By combining this novel genetic cross with rigorous *in vitro* resistance and fitness phenotyping, we have identified the genetic architecture that underlies decreased susceptibility to PPQ. While the DHA + PPQ ACT continues to be used as a frontline ACT, it is crucial to understand the genetic underpinnings of resistance.

## MATERIALS AND METHODS

### Preparation of the genetic crosses

We generated the crosses using FRG NOD huHep mice with human chimeric livers and *A. stephensi* mosquitoes as described by Vaughan et al*.* (42). Two individual recombinant pools were generated for each cross, using different cages of infected mosquitoes. To start each cross, gametocytes from both parental parasite strains were diluted to 0.5% gametocytemia in a human serum erythrocyte mix to generate infectious blood meals (IBMs). IBMs from each parent were mixed at equal proportions and fed to three cages of mosquitos (150 per cage).

We examined the mosquito infection rate and oocyst number per infected mosquito 7–10 days post-feeding. Fifteen mosquitoes were randomly picked from each cage and dissected under microscopy. Sporozoites were isolated from infected mosquito salivary glands, and 2–4 million sporozoites from each cage of mosquitoes were injected into three FRG huHep mice (one cage per mouse) intravenously. To allow the liver stage-to-blood stage transition, mice are infused with human erythrocytes 6 and 7 days after sporozoite injection. Four hours after the second infusion, the mice are euthanized and exsanguinated to isolate the circulating ring-stage *P. falciparum*-infected human erythrocytes. The parasites from each mouse constitute the initial recombinant pools of recombinant progeny for genetic mapping experiments. We maintained the initial pools in AlbuMAX-supplemented RPMI media; we genome sequenced aliquots from each pool to check allele frequencies from both parents.

### Library preparation and sequencing

We used the Qiagen DNA Mini Kit to extract and purify the genomic DNA and Quant-iT PicoGreen Assay (Invitrogen) to quantify the amount of DNA. For samples with less than 50 ng DNA obtained, whole-genome amplification (WGA) was performed before next-generation sequencing (NGS) library preparation. WGA reactions were performed following Nair et al. (62). Each 25-µL reaction contained at least 5 ng of *Plasmodium* DNA, 1× bovine serum albumin (New England Biolabs), 1 mM dNTPs (New England Biolabs), 3.5 µM of Phi29 Random Hexamer Primer, 1× Phi29 reaction buffer (New England Biolabs), and 15 units of Phi29 polymerase (New England Biolabs). We used a PCR machine (SimpliAmp, Applied Biosystems) programmed to run a “stepdown” protocol: 35°C for 10 min, 34°C for 10 min, 33°C for 10 min, 32°C for 10 min, 31°C for 10 min, and 30°C for 6 h then heating at 65°C for 10 min to inactivate the enzymes prior to cooling to 4°C. Samples were cleaned with AMPure XP Beads (Beckman Coulter) at a 1:1 ratio. We constructed NGS libraries using 50–100-ng DNA or WGA product following the KAPA HyperPlus Kit protocol with three cycles of PCR. All libraries were sequenced at 150-bp pair-end using Illumina Novaseq S4 or Hiseq X sequencers. We sequenced all bulk samples to a minimum coverage of 100×.

### Mapping and genotyping

We individually mapped whole-genome sequencing reads for each library against the *P. falciparum* 3D7 reference genome (PlasmoDB, release32) using the alignment algorithm BWA mem (http://bio-bwa.sourceforge.net/) under the default parameters. The resulting alignments were then converted to SAM format, sorted to BAM format, and deduplicated using picard tools v2.0.1 (http://broadinstitute.github.io/picard/). We used Genome Analysis Toolkit GATK v3.7 (https://software.broadinstitute.org/gatk/) to recalibrate the base quality score based on a set of verified known variants (43).

After alignment, we excluded the highly variable genome regions (subtelomeric repeats, hypervariable regions, and centromeres) and only performed genotype calling in the 21-Mb core genome [defined in reference (43)]. We called variants for each sample using HaplotypeCaller, and calls from every 100 samples were merged using CombineGVCFs with default parameters. Variants were further called at all sample levels using GenotypeGVCFs, with parameters: --max_alternate_alleles 6 --variant_index_type LINEAR --variant_index_parameter 128000 --sample_ploidy 2 -nt 20. We further filtered the variant calls by calculating the recalibrated variant quality scores (VQSR) of genotypes from parental parasites. Loci with VQSR less than 1 or not distinguishable between two parents were removed from further analysis. The variants in VCF format were annotated for predicted functional effect on genes and proteins using snpEff v4.3 (https://pcingola.github.io/SnpEff/) with 3D7 (PlasmoDB, release32) as the reference.

### Cloned progeny analysis

To identify unsuccessfully cloned progeny, we measured the multiplicity of progeny samples with F_WS_ (63), which is configured in moimix (https://github.com/bahlolab/moimix). Samples with *F*_WS_ < 0.9 were assumed to be non-clonal and were removed from further analysis. Allele frequencies across the genome were also plotted and manually inspected to detect further possible mixed infections.

We calculated IBD between clonal progeny and parents with hmmIBD (64) under default parameters. The proportions of shared IBD were used to determine relatedness among parental and progeny parasites and to identify genome regions inherited from each parent: (i) progenies with >90% shared IBD with either of the parents were assumed to result from selfing, (ii) progenies with <90% shared IBD with both parents were defined as recombinants, and (iii) recombinant progenies with >90% IBD against each other were defined as non-unique clones (65).

### Nanopore sequencing of parental parasites

We used an optimized Phenol:Chloroform DNA isolation protocol that generates 500-ng to 3-µg large-molecular weight genomic DNA from 40 mL of *in vitro* blood cultures. In brief, (i) short, fragmented DNA (<60 kb) is removed using the Small Read Eliminator Kit (Nanopore) and (ii) after DNA clean-up, we prepared sequencing libraries with the Ligation Sequencing Kit (Nanopore), which adds barcode to each sample. We used the Nanopore MinION Mk1C sequencer to generate long reads. We obtained 3.5 Gb (150× genome coverage) and 2.5 Gb (105×) of long-read sequencing data for Mal31 and KH004. Fifty percent of the data obtained comprises reads with length > 62 kb (N50 = 62 kb) with the longest read of 485 kb.

We mapped the nanopore long reads to *plasmepsin* gene sequences using *blastn* (https://blast.ncbi.nlm.nih.gov/Blast.cgi). Reads that cover all three *plasmepsin* genes and >10-kb flanking regions on both sides were then extracted and compared with the 3D7 reference genome to identify the repeat unit at *plasmepsin* genes.

### Plasmepsin II/III copy number identification

We analyzed mapped reads on the 67-kb extended loci of the *plasmepsin* genes (from 260,000 to 327,000 at chromosome 14), which included 28 kb upstream *plasmepsin* I and 28 kb downstream *plasmepsin* III. The number of reads mapped onto each position (coverage) was determined from the deduplicated BAM file using *bedtools* (https://bedtools.readthedocs.io/en/latest/). We visualized reads mapping to the 67-kb region using the IGV (Fig. S1A). The repeat unit of *plasmepsin* genes was confirmed by both IGV plot and nanopore long-read sequences. We calculated the *Plasmepsin* copy number using mean(coverage at repeat unit)/mean(coverage at extension regions).

### Linkage analysis

Linkage between the *pfcrt*, *pm2/3*, and *pfkelch13* loci was assessed by calculating D and D′ and utilizing Fisher’s exact test to determine significance. Association between *pfcrt* or *pfkelch13* alleles and *pm2/3* CN was assessed by binning multicopy *pm2/3* parasites and conducting Fisher’s exact test.

### Parasites used in this study

This study utilizes parents and progeny of the KH004 × Mal31 genetic cross generated by utilizing human liver-chimeric. From this cross, 104 unique recombinant progenies were recovered through limited dilution cloning at 0.3 cells per well. Individual wells containing parasites were identified through qPCR, and analysis of progeny relatedness and allele inheritance was performed based on the methods outlined by Button-Simons et al. (46). Methods for sequencing of progeny, construction of the physical map, and generation of visual recombination map are all based on the study by Button-Simons et al. (46). Analysis of allelic co-inheritance utilized the entire progeny set of 104 parasites, and phenotyping assays utilized 50 unique parasites, 48 progeny, and the 2 parental parasites.

### Parasite culture

Cryopreserved *P. falciparum* stocks of KH004 × Mal31 progeny and KH004-020-019-H9 and Mal31-9040-C11 parents were thawed and cultured in complete media consisting of 0.5% Albumax II- (Gibco, Life Technologies) supplemented RPMI 1640 with L-glutamine (Gibco, Life Technologies) with additional 50 mg/L hypoxanthine (Calbiochem, Sigma-Aldrich), 25 mM HEPES (Corning, VWR), 10 ug/mL gentamycin (Gibco, Life Technologies), and 0.225% sodium bicarbonate (Corning, VWR). Parasite cultures were maintained at 5% hematocrit in O+ red blood cells (RBC) (Interstate Blood Bank, Memphis, TN) in separate flasks and maintained consistent temperature (37°C) and atmosphere (5% CO_2_/5% O_2_/90% N_2_). Parasitemia of cultures was kept below 2%, and media changes were performed every 48 hours, corresponding to one intraerythrocytic development cycle.

### PfCRT structure analysis

The three-dimensional homology model of PfCRT was predicted using AlphaFold (66, 67) and accessed through the PDB database (6UKJ) (34). Additionally, the F′(ab) fragment used in 7G8 PfCRT cryo-EM structure elucidation (34) was removed from the structure. Protein mutagenesis and visualization of resistance-associated mutations were conducted using PyMol software (v2.5.4; Schrödinger, LLC). Comparison of PfCRT primary structure was conducted by performing an alignment between Dd2 (plasmoDB release32) and KH004 using Clustal Omega (68).

### Drug susceptibility assays

Prior to susceptibility assay setup, parasites were synchronized using a single layer of 70% Percoll (Sigma Aldrich) in 1× RPMI with 13.3% sorbitol in phosphate-buffered saline. Four hundred microliters of packed erythrocytes infected by a majority of schizont-stage parasites were resuspended in 2 mL of incomplete media, layered over the Percoll layer, and centrifuged (1,561 × *g* for 10 in, no brake). The top layer was removed to isolate late-stage schizonts, washed twice with incomplete media, resuspended at 5% hematocrit in complete media, and placed on a shaker for 4 hours at 37°C. Following the 4-hour incubation, parasitemia and stage were determined through flow cytometry by staining with SYBR Green I and SYTO 61 and analyzed on a Guava easyCyte HT (Luminex) after 50,000 events were counted. If cultures were >70% ring stage, they were diluted to 0.15% parasitemia at 2% hematocrit and set up in a 96-well plate at a volume of 150 µL per well. Within each assay plate, two technical replicates of each parasite were exposed to 10 concentrations of a twofold dilution series of PPQ, as well as both untreated parasite and uninfected RBC controls. Parasites were exposed to PPQ for 72 hours. Parasite density was determined using SYBR Green, and dose response curves were generated by plotting percent survival against the log of drug concentration. Analysis of IC_50_ and AUC was performed using Prism 9.0 (GraphPad). A nonlinear log inhibitor vs response 4 parameter dose response curve was generated based on percent survival. To generate the limited point curve, data points that made up the biphasic portion of the plot were excluded to allow for a sigmoidal-shaped curve, which was then used for IC_50_ calculations.

### PSA

PSA was set up as originally described (18) , with synchronized early ring stage parasites being exposed to a single 200-nM dose of PPQ for 48 hours. This protocol was modified to resemble the extended recovery RSA phenotype (69), in which samples are analyzed at 120 hours post-exposure instead of 72 hours. This extended timepoint has been shown to provide superior differentiation between resistant and sensitive parasites and allows for finer resolution between PPQ-S parasites. Parasite prevalence was measured through qPCR, and survival was calculated as fold change between untreated and treated samples for three technical replicates (three replicates within the plate as well as three independent qPCR reads) and at least three biological replicates per sample. Additionally, to ensure consistency between our resistance phenotypes, parasites from the same synchronization were used for both PSA and IC_50_/AUC susceptibility assays.

### Competitive growth assays

To determine the relative fitness levels between parasites, we used competitive growth assays between progeny of the KH004 × Mal31 cross and both parental parasites as a proxy for *in vitro* fitness. Parasitemia and stage of parasites were quantified using flow cytometry 15 hours after synchronization with a 70% Percoll gradient. Synchronized parasites were each adjusted to 0.5% parasitemia at 5% hematocrit and set up in a 1:1 ratio (1% total parasitemia) in 96-well plates as previously described in references (38, 40). Parasitemia was assessed (Giemsa-stained slides and microscopy) every 2 days, in accordance with one intraerythrocytic life cycle, adjusted to 1% parasitemia, and supplemented with fresh blood and media. Samples from each well were collected every 2 days upon sample dilution and stored at −80°C for genotyping. These competitive growth assays were maintained for a maximum of 40 days or until one parasite reached fixation (>90% of the co-culture). Parasites were deemed to have equivalent fitness levels if, by 40 days, neither parasite reached fixation.

To analyze relative parasite densities in each competition, six microsatellite markers were used for genotyping (Table S10) to differentiate parasites in co-culture. PCR amplification was performed using the Phusion Blood Direct PCR Kit (Thermo Fisher, cat #F547L) for 30 cycles, as previously described in reference (38). Amplified microsatellites were analyzed using the Applied Biosystems 3730xI DNA Analyzer (Thermo Fisher). Raw fragment analysis data were uploaded to the Thermo Fisher Connect platform and analyzed using the microsatellite analysis tool. The proportion of the two competing parasites in co-culture was determined by taking the ratio of fluorescent peak height associated with the corresponding PCR product size.

Due to the large number of competitive growth assays required to directly compete all parasites against each other, all parasites were competed against both parents. Additionally, a genetically diverse subset of parasites were competed against each other. Based on the outcomes of the progeny vs progeny competitions, an Elo rating system, which has previously been used for animal dominance hierarchies, was utilized to assign a quantitative score to each parasite based on their win/loss record while also taking into account the record of the parasite competed against (70). The package “EloRating” (54) in R Studio (2023.03.0+386) was used for assigning scores. To reduce the effect of competition order on ranking, the competitive outcomes file was randomized 1,000 times to generate 1,000 Elo scores, which were then averaged. The distribution of Elo ratings figure was created using Prism 9.0 (GraphPad).

To dissect the impact of *pm2/3* CNV alone on parasite fitness, we performed competitive growth assays, as described above, in the presence and absence of PPQ pressure between KH004 and FG305. KH004 and FG0305 are isogenic parasites which differ only in *pm2/3* CN (four copy vs one copy respectively). Parasites were exposed to PPQ for 3 days, with daily media changes reapplying fresh drug. Plates were washed three times with incomplete media to remove the drug after 3 days of consecutive exposure. Relative abundance of each parasite was determined via the average number of *pm2* copies present in the co-culture. The copy number was determined by the ∆Ct method [adapted from Ansbro et al. (71)] between *pm2* and *pfcrt* (single copy). Relative expression was calculated as 2^−∆Ct^. A pure culture of KH004 was grown alongside this competition to ensure that *pm2* CN was not changing within the timeframe of this experiment. Percent abundance was calculated by normalizing the average *pm2* CN in the KH004vFG0305 competition to the single KH004 sample on the same day.

### QTL analysis

Statistical analysis of the QTL data for this cross was performed using the computational methods previously described (72). Blood stages of *P. falciparum* are haploid; therefore, only two genetic classes are present for each locus. QTL mapping was performed using the “R/qtl” package (73) in R Studio. One thousand permutations of the trait values determined the 37%, 5%, and 1% genome-wide significance thresholds, and the strength of each linkage was expressed as a LOD score (74). The main QTL and corresponding mean trait values from these scans were used to obtain estimates of residual empirical thresholds to identify secondary loci that contribute to the phenotype of interest (74). After identifying QTLs associated with each phenotype, QTL interaction was tested using the scantwo function of the “R/qtl” package and was based on 1,000 permutations.

### Global haplotype analysis

Analysis of global *pfcrt* haplotypes was performed using publicly available sequences through the Pf7 data set (MalariaGEN) (12). The PPQ resistance status for this data set was inferred based on *plasmepsin II/III* amplification; therefore, all samples labeled as PPQ-R are assumed to express more than one copy of these genes.

## Data Availability

All data needed to evaluate the conclusions in the article are present in the article and/or the supplemental material. All raw sequencing data have been submitted to the NCBI Sequence Read Archive (SRA, https://www.ncbi.nlm.nih.gov/sra) under the project number PRJNA524855. Additional data related to this paper may be requested from the authors. The code used in analysis and data analyzed are available at GitHub through the following links: https://github.com/FerdigLab/KH004xMal31_Genetic_Cross (K.A.B-.S.) and https://github.com/emilyli0325/PM-CNV-in-PPQ (X.L.).

## AFTER EPUB

*[This article was published on 24 June 2024 with an error in the References. The References were corrected in the current version, posted on 28 June 2024.]*

## SUPPLEMENTAL MATERIAL

Supplemental Figures mbio.00805-24-s0001.pdf Figures S1-S5.

Supplemental Tables mbio.00805-24-s0002.xlsx Tables S1-S10.

ASM does not own the copyrights to Supplemental Material that may be linked to, or accessed through, an article. The authors have granted ASM a non-exclusive, world-wide license to publish the Supplemental Material files. Please contact the corresponding author directly for reuse.
