# Supplementary material for: A Plasmodium falciparum genetic cross reveals the contributions of pfcrt and plasmepsin II/III to piperaquine drug resistance
Source: mBio. 2024 Jun 24;15(7):e00805-24. doi: 10.1128/mbio.00805-24 (PMC11253641; doi:10.1128/mbio.00805-24)
Supplement: Supplemental Figures — Figures S1-S5. [file mbio.00805-24-s0001.pdf]

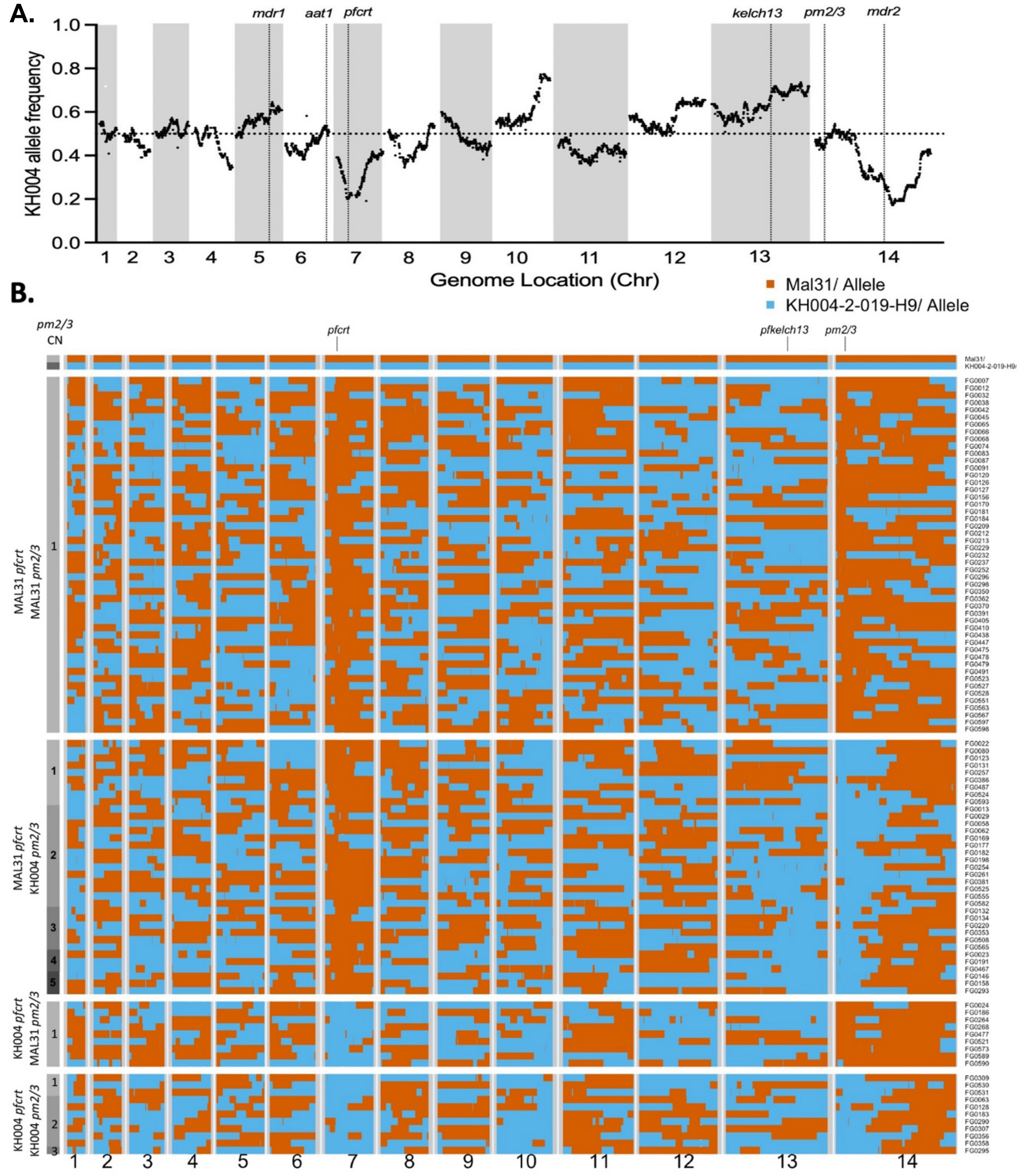

Figure S1: Inheritance breakdown of KH004xMal31 genetic cross. *A) From this cross, we see two strong skews in inheritance on chromosomes 7 and 14, both favoring the Mal31 allele. This selective inheritance has previously been observed in other genetic crosses between Southeast Asian and African parasites. The chromosome 7 peak is centered around pfcrt and therefore represents a strong selection against the inheritance of the KH004 allele at these positions. A similar negative selection against a set of loci on chromosome 14, likely driven by arps10, has previously been identified in additional genetic crosses (40, 46). B) Aside from the selective inheritance on chromosomes 7 and 14, we observe no significant co-inheritance patterns between pfcrt, pm2/3, or kelch13. Each horizontal line represents one unique recombinant parasite, with the Mal31 and KH004 parental parasites being the first two rows respectively. Inheritance of the KH004 allele is denoted in blue and the Mal31 allele in orange.*

```
PfDd2_070013200      MKFASKKNNQKNSSKNDERYRELDNLVQEGNGSRLGGGSCLGKCAHVFKLIFKEIKDNIF      60
KH004-019-H9         MKFASKKNNQKNSSKNDERYRELDNLVQEGNGSRLGGGSCLGKCAHVFKLIFKEIKDNIF      60
                     ************************************************************

PfDd2_070013200      IYILSIIYLSVCVIETIFAKRTLNKIGNYSFVTSETHNFICMIMFFIVYSLFGNKKGNSK      120
KH004-019-H9         IYILSIIYLSVCVIETIFAKRTLNKIGNYSFVTSETHNFICMIMFFIVYSLFGNKKGNSK      120
                     ************************************************************

PfDd2_070013200      ERHRSFNLQFFAISMLDACSVILAFIGLTRTTGNIQSFVLQLSIPINMFFCFLILRYRYH      180
KH004-019-H9         ERHRSFNLQFFAISMLDACSVILAFIGLTRTTGNIQSFVLQLSIPINMFFCFLILRYRYH      180
                     ************************************************************

PfDd2_070013200      LYNYLGAVIIVVTIALVEMKLSFETQEENSIIFNLVLISSLIPVCFSNMTREIVFKKYKI      240
KH004-019-H9         LYNYLGAVIIVVTIALVEMKLSFETQEENSIIFNLVLISSLIPVCFSNMTREIVFKKYKI      240
                     ************************************************************

PfDd2_070013200      DILRLNAMVSFFQLFTSCLILPVYTLPFLKELHLPYNEIWTNIKNGFACLFLGRNTVVEN      300
KH004-019-H9         DILRLNAMVSFFQLFTSCLILPVYTLPFLKELHLPYNEIWTNIKNGFACLFLGRNTVVEN      300
                     ************************************************************

PfDd2_070013200      CGLGMAKLCDDCDGAWKTFALFSFFSICDNLITSYIIDKFSTMTYTIVSCIQGPATAIAY      360
KH004-019-H9         CGLGMAKLCDDCDGAWKTFALFSFFSICDNLITSYIIDKFSTMTYTIVSCIQGPATAIAY      360
                     ************************************************************

PfDd2_070013200      YFKFLAGDVVIEPRLLDFVTLFGYLFGSIIYRVGNIILERKKMRNEENEDSEGELTNVDS      420
KH004-019-H9         YFKFLACDVVIEPRLLDFVTLFGYLFGSIIYRVGNIILERKKMRNEENEDSEGELTNVDS      420
                     ****** *****************************************************

PfDd2_070013200      IITQ        424
KH004-019-H9         IITQ        424
                     ****
```

Figure S2: PfCRT sequence comparison between KH004 and Dd2. *PfCRT sequence for KH004 was aligned with Dd2 (acquired from PlasmoDB) using Clustal Omega (68). These two parasites differ at pfcrt by a single G367C substitution (red). Additionally, KH004 has one SNP (401883C>T) in the intergenic region upstream of pfcrt which is also found in Dd2. The similarity in pfcrt sequences between these two parasites strengthens the association between the G367C substitution and decreased PPQ sensitivity.*

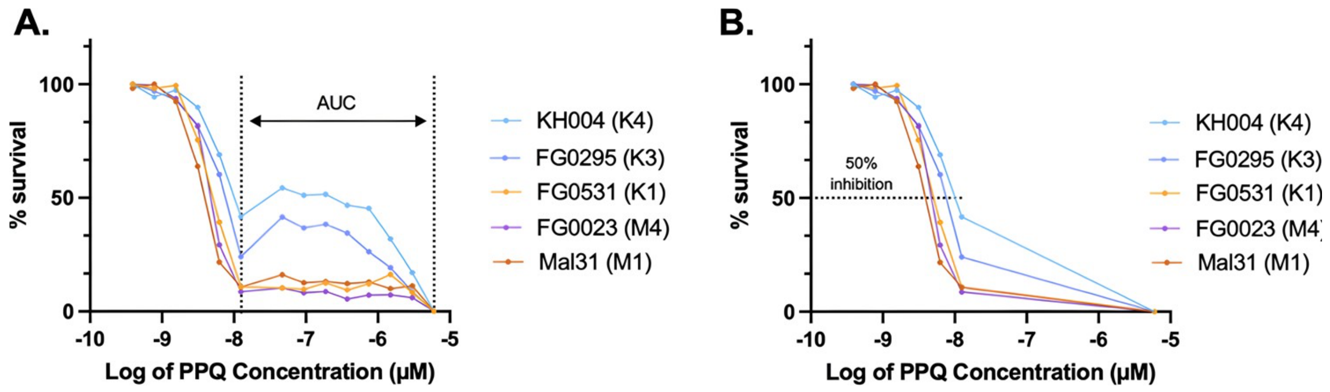

Figure S3: Shape of dose-response curve changes based on pfcrt and pm2/3 genotype. *Traditional IC_{50} measurements have been a challenge with PPQ due to the biphasic dose-response curve observed in PPQ-R parasites. A) We have found inheritance of the KH004 pfcrt allele and multiple copies of pm2/3 are required for producing a biphasic curve. Progeny inheriting the Mal31 pfcrt allele do not produce a biphasic curve, regardless of pm2/3 CNV. Additionally, all parasites expressing a single copy of pm2/3 produce a sigmoidal curve. To account for these various dose- response curve shapes, AUC is used as a measure of resistance, with a greater AUC indicating decreased PPQ susceptibility. B) By excluding the points in the dose-response curve which are responsible for the biphasic portion of the curve, we can eliminate the secondary peak in the curve and measure an IC_{50} independent of original curve shape (LP-IC_{50}). Key next to parasite name indicates pfcrt allele (KH004 or Mal31) as well as copy number of pm2/3.*

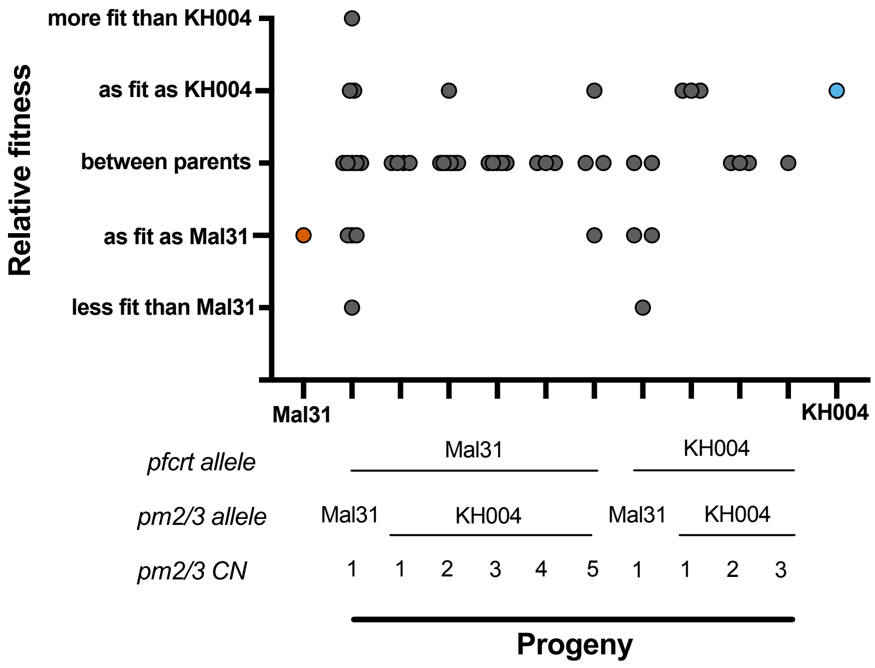

Figure S4: Competitive growth assessment between progeny and parents. *Head-to-head competitive growth assays were set up between progeny and parents. Parasite fitness was determined based on win-lose-tie outcome of competition against both KH004 and Mal31. Progeny are displayed based on inherited genotype at pfcrt and pm2/3, with Mal31 (orange) and KH004 (blue) being specifically denoted. The relative fitness of the majority of progeny from the KH004×Mal31 genetic cross fall in between the two parents, with no significant association between relative fitness and genotype of either pfcrt or pm2/3.*

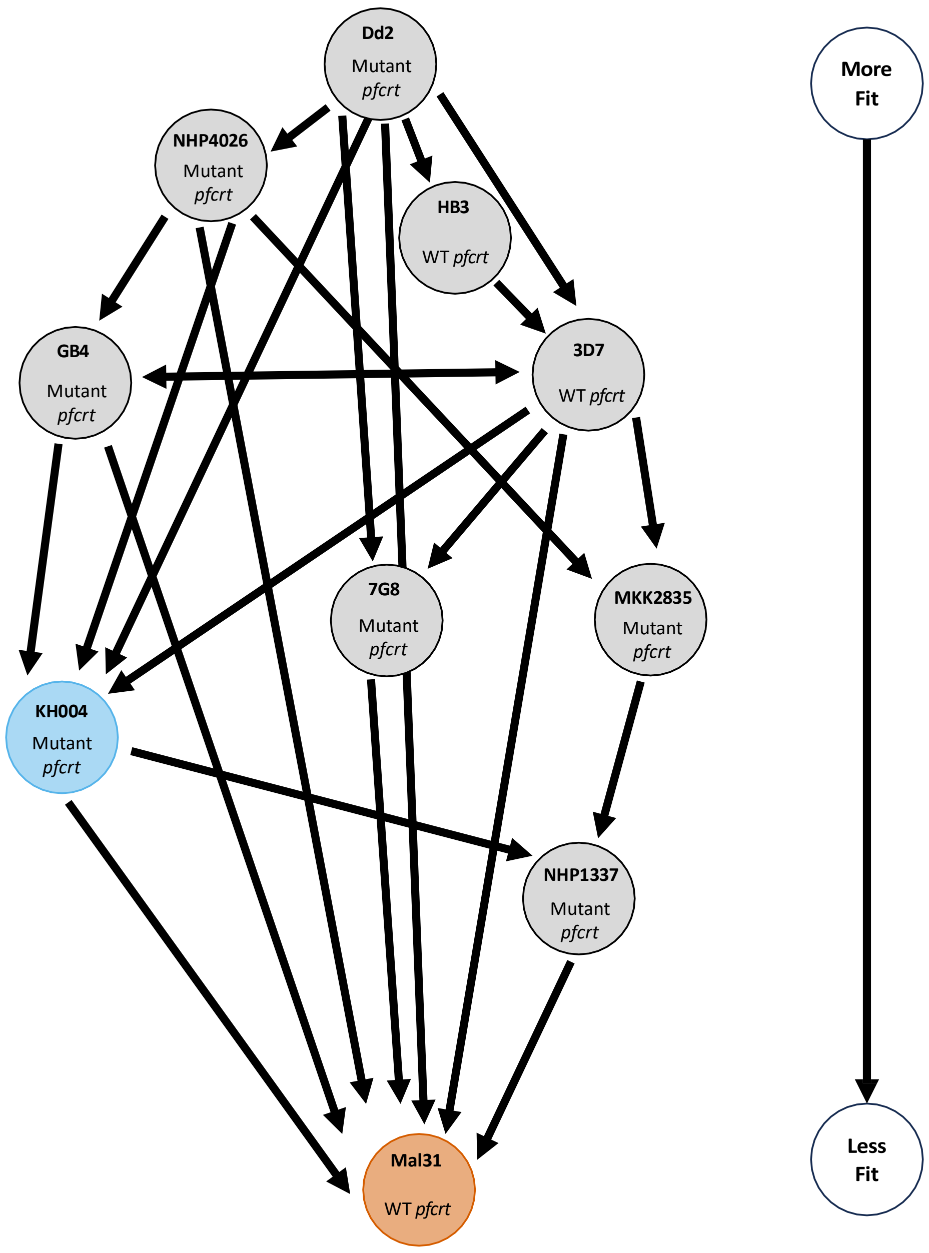

Figure S5: Fitness hierarchy among genetic cross parents. *Head-to-head competitive growth assays were performed between KH004, Mal31, and the parents of previously performed genetic crosses to construct a relative fitness hierarchy. Arrows connecting parasites indicate direct outcomes of competitions. Horizontal two-headed arrow indicates a competitive fitness assay resulting in a tie. While KH004 displays the highest level of fitness in this cross, it is less fit than many other isolates, regardless of genotype and resistance phenotype.*
